# 500. Impact of an iron chelator on *in vitro* and *in vivo* growth of *A. baumannii*

**DOI:** 10.1093/ofid/ofac492.557

**Published:** 2022-12-15

**Authors:** Brianna Eales, Bing Bai, Vincent Tam

**Affiliations:** University of Houston, Pasadena, Texas; The 6th Affiliated Hospital of Shenzhen University Health Center, Shenzhen, Guangdong, China; University of Houston, Pasadena, Texas

## Abstract

**Background:**

*Acinetobacter baumannii* (AB) has become multidrug-resistant in recent years and currently there is no consensus on the optimal treatment for these infections. To combat this urgent threat, novel treatment options are needed to minimize spread and lower mortality rates. Nutrient metals (e.g., iron) are essential to the metabolic functions of AB. If the bioavailability of essential nutrients is restricted, this can cause a bacterial stress response and alter their ability to multiply.

**Methods:**

The objective of the study was to examine the impact of iron chelation on the growth of AB *in vitro* and *in vivo*. MDR AB bloodstream isolates (n=4) were recovered from unique patients between 2011 and 2018. Clonal diversity was ascertained by Fourier Transform Infrared Spectroscopy. *In vitro* bacterial densities were measured longitudinally over 20-hours to determine the growth profile. Isolates were started at a baseline concentration of approximately 5.5 log CFU/mL in tryptic soy broth. Variable amounts of an iron chelating agent (deferiprone, 0.2-2mM) were added to create a concentration gradient. *Galleria mellonella* larvae (100-150mg) were inoculated with approximately 5 log CFU of an isolate, with and without deferiprone (10mM). Quantitative culture was used to ascertain the bacterial burden of aggregate larvae (n=10) immediately and 4h post infection. Additional infected larvae (n=20 in each group) were incubated at 35°C and monitored hourly for 24h.

**Results:**

Time to mortality was compared using Kaplan-Meier survival analysis and log-rank test. Increasing concentrations of the chelating agent caused a transient and concentration-dependent hindrance of *in vitro* bacterial growth, compared to the no-treatment group. Bacterial burden immediately post-infection of the control and treatment group were comparable at 6.4 log CFU/g. After 4 hours the bacterial burden was much higher in the control group than the treatment group, 8.7 and 6.7 log CFU/g respectively. A trend of delayed mortality was shown in the iron chelator group (median survival 6.5 vs 10 hours, p=0.15).

**Conclusion:**

These results support that micro-nutrient limitation has the potential to be a novel approach for treating high-risk infections due to MDR AB. Future studies are warranted to further define its optimal place in therapy.

**Disclosures:**

**All Authors**: No reported disclosures.

